# Chemicals having estrogenic activity can be released from some bisphenol a-free, hard and clear, thermoplastic resins

**DOI:** 10.1186/1476-069X-13-103

**Published:** 2014-12-04

**Authors:** George D Bittner, Michael S Denison, Chun Z Yang, Matthew A Stoner, Guochun He

**Affiliations:** CertiChem, Inc, 11212 Metric Blvd, Suite 500, Austin, TX 78758 USA; Department of Neuroscience, The University of Texas, Austin, TX 78758 USA; Department of Environmental Toxicology, University of California, Davis, CA 95616 USA

**Keywords:** BG1Luc, Bisphenol A, Tritan, Estrogenic activity, MCF-7, Thermoplastic resins

## Abstract

**Background:**

Chemicals that have estrogenic activity (EA) can potentially cause adverse health effects in mammals including humans, sometimes at low doses in fetal through juvenile stages with effects detected in adults. Polycarbonate (PC) thermoplastic resins made from bisphenol A (BPA), a chemical that has EA, are now often avoided in products used by babies. Other BPA-free thermoplastic resins, some hypothesized or advertised to be EA-free, are replacing PC resins used to make reusable hard and clear thermoplastic products such as baby bottles.

**Methods:**

We used two very sensitive and accurate *in vitro* assays (MCF-7 and BG1Luc human cell lines) to quantify the EA of chemicals leached into ethanol or water/saline extracts of fourteen unstressed or stressed (autoclaving, microwaving, UV radiation) thermoplastic resins. Estrogen receptor (ER)-dependent agonist responses were confirmed by their inhibition with the ER antagonist ICI 182,780.

**Results:**

Our data showed that some (4/14) unstressed and stressed BPA-free thermoplastic resins leached chemicals having significant levels of EA, including one polystyrene (PS), and three Tritan™ resins, the latter reportedly EA-free. Exposure to UV radiation in natural sunlight resulted in an increased release of EA from Tritan™ resins. Triphenyl-phosphate (TPP), an additive used to manufacture some thermoplastic resins such as Tritan™, exhibited EA in both MCF-7 and BG1Luc assays. Ten unstressed or stressed glycol-modified polyethylene terephthalate (PETG), cyclic olefin polymer (COP) or copolymer (COC) thermoplastic resins did not release chemicals with detectable EA under any test condition.

**Conclusions:**

This hazard survey study assessed the release of chemicals exhibiting EA as detected by two sensitive, widely used and accepted, human cell line *in vitro* assays. Four PC replacement resins (Tritan™ and PS) released chemicals having EA. However, ten other PC-replacement resins did not leach chemicals having EA (EA-free-resins). These results indicate that PC-replacement plastic products could be made from EA-free resins (if appropriate EA-free additives are chosen) that maintain advantages of re-usable plastic items (price, weight, shatter resistance) without releasing chemicals having EA that potentially produce adverse health effects on current or future generations.

## Background

### Estrogenic activity (EA)

Chemicals that mimic or antagonize the *in vitro* and/or *in vivo* actions of naturally occurring estrogens such as 17β-estradiol (E2) are typically defined as having estrogenic activity (EA) or anti-estrogenic activity, and effects on estrogen signaling represent the most common and best studied endocrine disruptor activity [1–4]. Xenobiotic chemicals exhibiting EA often interact with more than one estrogen receptor (ER) subtypes [3–6] and can produce many biological and adverse health effects in mammals, such as early puberty in females, reduced sperm counts, altered functions of reproductive organs, obesity, altered sex-specific behaviors, and increased rates of some breast, ovarian, testicular, and prostate cancers [1–9]. Fetal, newborn, and juvenile mammals are reported to be particularly sensitive to chemicals having EA, and effects have been observed at very low doses. Such adverse health effects are often first detected in the adult mammal [4, 8–11]. Many of the effects observed in other mammals would also be expected to be produced in humans since basic endocrine mechanisms are highly conserved across all classes of vertebrates [3, 12].

### Materials and processes in plastics production

Though outwardly simple, a plastic item such as a baby bottle is the end product of a wide range of materials and processes [13–21]. There are two principal classes of plastics, thermosets and thermoplastics [20, 21]. These two types of resins are the most prevalent plastics in the U.S. for food and beverage packaging, due to their low cost, high performance, and ease of processing. Baby bottles usually have component parts from both thermoset and thermoplastic resins [20, 21]. Thermoset polymer resins can not be re-melted and remolded, e.g., phenolics, epoxies, and polyurethanes. Thermoplastic polymers can be melted and remolded multiple times. to make products, some of which are flexible and not transparent, such as those made from polypropylene (PP), or polyethylene (PE) resins. Other thermoplastic resins are used to make hard and clear products typically for single use only such as polyethylene terephthalate (PET, aka PETE) resins. Yet-other thermoplastic resins such as cyclic olefin copolymer (COC), polystyrene (PS) and polycarbonate (PC) resins are used to manufacture hard, clear and very durable (re-usable) products such as baby bottles and food storage containers.

**Figure 1:**
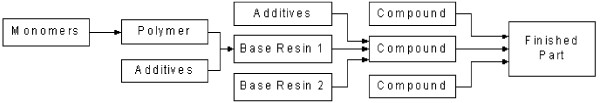
**Schematic diagram of the manufacturing process to make a plastic part.**

Thermoplastic resins are made by polymerizing a specific monomer or monomers in the presence of catalysts and other chemicals (additives) at high temperatures and pressures into a high molecular weight chain, i.e., a thermoplastic polymer (Figure 1). The resulting polymer is mixed with small quantities of other additives (antioxidants, plasticizers, clarifiers, etc.) and melted, mixed, and extruded to form a base thermoplastic resin as a powder, pellet or sheet (plaque) [20, 21]. Such base resins are then either used as-is (e.g., bisphenol A (BPA)-based PC resins) or, more commonly, mixed with other resins, additives such as antioxidants, colorants, and/or extenders to form plastic compounds (e.g., polymer blends, pre-colored polymers). Plastic products are then made by conversion processes (e.g., molding) using one or more plastic resins or compounded resins (compounds) to form a plastic part that is typically subjected to finishing processes that utilize other additives such as inks, adhesives, etc., to make the finished product [20, 21].

For simplicity, Figure 1 shows just one set of inputs in a process flow to make thermoplastic base resins and compounds; actual finished parts often have even more polymers, additives, or other chemicals. That is, each plastic part is a rather unique combination of (typically) 10 or more chemicals, and a plastic product with many parts (e.g., a baby bottle) often consists of 30–100 chemicals [20, 21]. Any of these chemicals might have EA [8, 16, 17] and leach from the final product because polymerization is almost always incomplete, leaving residual unincorporated monomers and/or because most additives (e.g., antioxidants) are not chemically part of the polymeric structure [20, 21]. Various stresses such as UV light, microwave radiation, and moist heat can also cause chemical changes in resins or plastic products [20, 21], possibly converting EA-free monomers or additives into chemicals exhibiting EA [16, 17]. Accounting for such factors may individually appear obvious or mundane, but are essential to producing EA-free resins or plastic parts, and to our knowledge have not been explicitly considered individually, much less in aggregate, by any publication of which we are aware other than [16, 17] for products. Furthermore, if a resin or product contains chemicals that have EA, then the final finished resin or product will almost-certainly leach chemicals having EA, since no additive or process known to us could prevent their leaching, sometimes especially if stressed [17]. All previous relevant publications [16, 17] have examined plastic products, not the resins used to make such products.

A small subset (especially BPA-based PC resins) of all types of thermoplastic resins have often been chosen to make reusable, hard and clear plastic items to contain food, including items used by babies [4, 8–13]. The US FDA has advised against the use of PC reusable thermoplastic resins synthesized by polymerizing BPA, a chemical with EA [13]. Similar use of PC resins has also been limited or banned in some other jurisdictions such as Minnesota, Canada and the European Union [13]. However, other non-BPA-containing thermoplastic resins used to make hard and clear reusable plastic products, such as glycol-modified polyethylene terephthalate (PETG), PS, nylon, cyclic-olefin-polymer (COP), COC resins, co-polyester Tritan™ or Bisphenol S (BPS) resins might also contain and release chemicals that have EA [14–16]. In fact, BPS and BPS analogs have recently been shown to exhibit EA [14, 15], and as discussed above, products made from such resins would be expected to leach BPS and have detectable EA [16, 17]. The other resins listed above have not been tested for release of chemicals exhibiting EA. Given that BPA-free thermoplastic resins are now widely used to replace PC resins in a variety of commercial and consumer products, including food storage containers, water bottles, baby bottles, and sippy cups [13, 18, 19], confirmation that chemicals with EA do not leach from BPA-free PC-replacement resins is critically needed.

### EA in hard and clear, reusable, BPA-free thermoplastic resins

In this paper, we describe the first study of which we are aware that assesses the release of chemicals having EA from plastic resins that were either unstressed or subjected to different stressors. We tested fourteen unstressed and stressed (microwaved, autoclaved, or ultraviolet (UV) irradiated) thermoplastic resins that could substitute for BPA-based resins, e.g., COC, COP, nylon, PETG, PS, and Tritan™ resins, to make hard and clear, reusable, plastic products. We examined each BPA-free, PC-substitutable, resin unstressed and/or stressed by microwaving, autoclaving or exposure to short and/or long wavelength ultraviolet radiation. The stresses were employed to simulate some aspects of the wear and tear (aging) of typical use [16, 17]. Different stresses might increase (or decrease) chemical leaching and some stresses might produce additional leachable chemicals having EA. Because chemicals having EA may be more- or less-polar and are best extracted by a solvent that better matches their polarity [20, 21], unstressed or stressed samples of plastic resins were extracted using more-polar (water, saline) or less-polar solvents (EtOH).

EA in resin leachates was determined using two *in vitro* mammalian cell-based bioassays, one that measures ER-dependent gene expression (BG1Luc assay: [22]) and another that measures ER-dependent cell proliferation (MCF-7 assay: [16, 23]). The role of the ER in the EA response was confirmed by suppression of the ER response with the pure estrogen receptor antagonist ICI 182,780 (ICI) [24]. The results showed that chemicals with EA were released from four BPA-free thermoplastic resins (a PS and three Tritan™ resins) by polar and/or non-polar solvents when unstressed and/or stressed, but especially when stressed with various UV sources, including sunlight. Products made from these resins would be expected to release chemicals having EA, no matter what other chemicals might be added to the resin to make a final product. Thus, BPA-free does not necessarily mean EA-free. Ten other thermoplastic resins of types COC, COP, nylon, and PETG did not release chemicals having detectable EA into more-polar and/or less-polar solvents, whether or not they were stressed. Production of EA-free thermoplastic products using these EA-free resins (e.g. COC, COP, PETG) would be possible if no EA-containing additives were used and could explain our previous finding [17] that EA-free plastic products can be found made from COC, COP, and PETG resins.

## Methods

Fourteen resins were received by CertiChem (CCi) in 2008–2012 and by University of California at Davis (UCD) in 2012. Analyses at CCi focused on assessing release of chemicals having EA from many different stressed and unstressed resins, especially three Tritan™ resins, although relatively fewer assays were performed on unstressed Tritan™ resins. In contrast, UCD focused on EA release from three unstressed Tritan™ resins. The three Tritan™ resins tested were from a family of similar resins that are synthesized from the same monomers and probably the same additives, but in different concentrations. Some resins may even been the same (e.g. EX401 and TX201), but are packaged and marketed differently. At CCi, each resin was subjected to various (not necessarily the same) combinations of extraction solvents and stresses and EA was determined by two different assays, MCF-7 and BG1Luc (see below). CCi sometimes stopped testing a resin if a particular type of extraction solvent or stress clearly showed that the resin leached significant levels of chemicals having EA. In other cases, a resin was run many times as a positive control for other studies and all such data are reported herein. That is, the aim of this study was not to perform an exhaustively complete set of responses, using the same number of replicate assays for all possible stresses and extraction conditions for each resin, but rather to survey a larger sample of PC-replacement resins and assess whether they released chemicals with EA. Resins were sometimes tested once each for three different assay conditions, rather than two or more times with one given assay condition.

### Protocols to stress plastic resins

Prior to applying stresses at CCi, resin pellets were compression molded at 230°C into 4 × 4 × 1/64 inch thick plaques. Heat and moisture stresses were obtained by sealing plaques in individual crimped packets of aluminum foil and placing them into a Tuttnauer autoclave set at 134°C for 8 minutes.

Microwave stresses were obtained by placing samples into glass beakers and microwaving them in a 1200 W oven set to “high” for two minutes, allowed to sit for 30 minutes, and the cycle repeated 10 times. Alternatively, samples were placed in EA-free polypropylene tubes, microwaved on “high” setting for three minutes with a resting period of 30 minutes and this cycle repeated 5 times. Both methods produced very similar results.

Several methods of UV stressing were carried out as previously described [16, 17]:

1. Long wavelength (315-400 nm) UVA stresses: plaques were placed in a Q-Lab QUV unit containing UVA-340 nm bulbs (intended to simulate sunlight between 295 nm and 365 nm) for 80 hours at 45–50°C with no condensation.
2. Short wavelength (100-280 nm) UVC stresses: plaques were placed onto a piece of aluminum foil in a Labconco Biosafety hood approximately 24” from a germicidal fluorescent light (maximum intensity wavelength of 254 nm) for 24 hours.
3. Natural sunlight stresses: plaques were placed individually between a quartz glass plate on top and aluminum foil on a porcelain plate below. The two plates were clamped together using binder clips. To control for heat versus sunlight effects, some of these plaques were wrapped in thick aluminum foil. These plaques were placed on the roof of CCi’s facility for 1–14 days in summer.

Note that UV radiation in sunlight is often classified by wavelength [25] as UVC (100–280 nm), UVB (280–315 nm), and UVA (315–400 nm). Most UVC wavelengths do not reach the earth’s surface. However, UVC wavelengths are used in some germicidal UV devices, e.g., to sterilize baby bottles. Visible wavelengths are 400–750 nm.

### Extracts of plastic resins/plaques

At CCi, unstressed or stressed plaques described above were cut into ~4 × ~4 mm pieces and 2.0-5.0 grams of these cut resin pieces were added to sterile glass test tubes and placed under a germicidal UV light for 30 minutes to sterilize the samples before adding an extraction solvent to a final concentration of 1.0 g of resin/ml. This brief exposure to UV does not alter release of chemicals having EA from plastics [16]. The extraction solvents used were a saline-based solution (saline: Roswell Park Memorial Institute RPMI-1640 medium without Phenol red), 100% EtOH, 10 or 50% aqueous EtOH, or distilled water. Some samples were extracted for 72 hours at 37°C in a static incubator; the majority of samples were extracted at 40°C for 240 hours in an incubator shaker. EtOH extracts were concentrated 10× by evaporation and then diluted 100× with estrogen-free-medium (EFM) with the highest resin concentration at 0.1 g of resin/ml. Saline extracts were diluted 1:1 with 2× EFM and then diluted 1-4× with EFM so that the highest resin concentration was 0.125 g resin/ml. Estrogen-free medium is actually estrogenic-activity (EA)-free medium and it is made “essentially free” of EA by using phenol-red-free basal medium supplemented with dextran-coated/charcoal stripped fetal bovine serum (FBS).

Unstressed resin pellets were used as received at the University of California, Davis (UCD). Unstressed resin pellets (6.0 g) were extracted with 3.5 ml 100% EtOH in a baked glass tube with Teflon-lined cap for three days at room temperature in the dark. The extract solution was transferred to a second baked glass tube, and then evaporated to dryness. The residue was dissolved in 40 μl DMSO, vortexed, and diluted with 1960 μL of EFM. This protocol produced stock solutions of ~3.0 g resin/ml in medium containing 1% DMSO. The stock solutions were serially diluted such that the highest test concentration was 1.5 g resin equivalent/ml. The highest g resin concentration/ml for BG1Luc assays run at UCD was 4–15 times higher than the highest g resin concentration/ml for MCF-7 or BG1Luc assays run at CCi.

We used more than one extraction method for many reasons. First, there are no standards yet devised for such extracts. Second, our data in [16, 17] and this paper clearly showed that more than one extraction solvent was needed to better detect hydrophilic versus hydrophobic chemicals in leachates. Third, different extraction solvents require different dilution protocols. That is, isotonic saline extracts can be tested without dilution, but distilled water or various EtOH extracts must be diluted to a starting EtOH concentration of no more than 1% EtOH. [Higher EtOH concentrations kill cells in cell-based *in vitro* assays such as the MCF-7 and BG1 assays]. Third, evaporation of the solvent concentrates the extracted chemicals, but would lose chemicals that are more volatile than the solvent. EtOH extracts were always concentrated, in part because EtOH extracts had to be diluted (see above) while saline extracts did not have to be diluted and hence were often not concentrated. In brief, using a single protocol would have affected the results, unfortunately by hiding/not finding many chemicals that had EA in leachates.

### Materials and supplies

Both cell types were grown and maintained in polystyrene T-75 flasks (BD Falcon, BD Biosciences, San Jose, CA, cat#353136) and polystyrene T-25 flasks (CytoOne, USA Scientific, Ocala, FL, cat#CC7682-4825). Media and medium supplements (RPMI -1640 Medium, DMEM, FBS, nonessential amino acids, L-glutamine, penicillin, streptomycin) were purchased from Invitrogen (Grand Island, NY, USA). Insulin was purchased from Sigma (St. Louis, MO, USA).

MCF-7 cells were seeded into 96-well flat bottom PS polystyrene plates (BD Falcon, cat#353075) and BG1Luc cells were seeded into 96-well white wall/clear bottom plates (Greiner Bio-One, Monroe, NC, cat#655098). Ethanol of 100% purity was purchased from OmniPur, EMD-Millipore, Billerica, MA, Acros Organics/Fisher Scientific, Pittsburgh, PA or Sigma-Aldrich, St. Louis, MO. Water was distilled on-site in an all-glass system and collected directly into glass before use in extractions performed in borosilicate glass tubes. ICI was obtained from Tocris Bioscience (Minneapolis, MN, USA).

### BG1Luc Assays

The BG1Luc4E2 cell line (referred to here as BG1Luc) is a human ovarian cancer cell line that responds to estrogenic chemicals with the induction of firefly luciferase [26], and has been approved as a screening method for estrogenic chemicals by OECD, EPA, and ICCVAM/ NICEATM [22]. BG1Luc cells were maintained in cell culture medium, and then placed in EFM for 3 (CCi) or 5 (UCD) days. Standard BG1Luc cell culture maintenance medium is complete growth medium containing phenol red, FBS and various additives and thus contains estrogen and chemicals with EA that facilitate cell growth. The EFM used for detection and analysis of EA using the BG1Luc cells was of two varieties: one EFM was identical to the MCF-7 EFM (see below), the other EFM used a different phenol-red-free basal medium (DMEM) supplemented with 10% dextran-coated/charcoal-stripped FBS.

Acclimated cells were seeded at 10,000 (CCi) or 70,000 (UCD) cells per well in 100 μL EFM in 96-well plates for 24 h, followed by a 24 ± 6 h incubation with test extracts in triplicate. The use of slightly different protocols by the two laboratories meant that quantitative values for %RME2 could not be directly compared, but insured that qualitative conclusions (the presence or absence of EA in sample leachates) did not depend upon one specific protocol for the BG1Luc assay used by one laboratory. Cytotoxicity was assessed by microscopic inspection as previously described [17, 22, 23, 27]. Cell culture medium was aspirated, cells were lysed with 1% Triton X-100, 10% glycerol, 2 mM EGTA, and 1 mM DTT. Luciferase was then measured using an automated microplate luminometer (Tristar, Berthold) with the Promega Luciferase Assay System as previously described [17, 26, 27].

### MCF-7 Assays

We used an MCF-7WS8 cell line, a human breast cancer cell line, in a robotic version [16, 17, 23] of the MCF-7 cell proliferation assay (aka E-screen assay) that has been used for decades to reliably assess EA [28, 29]. The assay has been undergoing validation for international use by ICCVAM/ NICEATM and has been nominated for validation by OECD. Chemicals with EA activate the ERs and ER-dependent transcription of estrogen-responsive genes, which leads to proliferation of MCF-7 cells. In brief, each test chemical or extract at each concentration was added in triplicate or quadruplicate to 96-well plates containing MCF-7 cells in EA-free culture media. MCF-7 EA-free culture medium is phenol-red-free RPMI 1640 medium supplemented with antibiotic/antimycotic solution and L-glutamine, 1% dextran-coated/charcoal stripped FBS, and 4% dextran-coated/charcoal stripped FBS.

After six days of exposure, MCF-7 cells were lysed with 1:5 (vol/vol) of 0.16% acetaldehyde/20% perchloric acid. The amount of DNA/well, an indication of cell numbers, was assayed using a microplate modification of the diphenylamine assay as previously reported [16, 17, 23]. Cytotoxicity was assessed as described above for BG1Luc assays and as previously described for MCF-7 assays [16, 17, 23].

### Calculation of EA

The EA of extracts was calculated as the relative maximum %E2 (%RME2, a measure of response amplitude), i.e., a percentage of the maximum DNA/well (MCF-7 assays) or relative luminescence (BG1Luc assays) produced by an extract at any dilution relative to the maximum agonist effect produced by E2 [16, 23, 27]. Aliquots of the highest concentration of an extract were diluted four to eight times to produce up to eight test concentrations. Each test chemical or extract at each concentration was added in triplicate or quadruplicate to 96-well plates containing MCF-7 cells in EA-free culture media. Both a vehicle control (VC) and “sham” control (SC), also called a “method blank”, were run in triplicate or quadruplicate in each experiment. The VC was the vehicle solvent used for that particular assay. The SC was the vehicle solvent taken through all steps that were used to assay the test sample/test extract. The VC was set to 0% RME2. For the MCF7 assay, cell proliferation responses were normalized by the DNA response of the vehicle control according to Equation 1 [16, 23]:
1

For the BG1 assay, %RME2 was similarly calculated by subtracting the VC RLU (relative luminescence units) from test extract RLU, and then normalized by dividing by the VC-adjusted highest E2 RLU response. The maximum E2 response was determined by a concentration-response curve run in triplicate for 8 – 12 E2 concentrations for each assay. The maximum E2 response was set to 100% RME2 and the VC to 0%RME2. The concentration-response curve for EA of a test substance or extract was plotted with log M or log g/mL test concentrations respectively, on the X-axis and %RME2 on the Y-axis.

Typical values for SC were 0% ±10% RME2. The %RME2 of a sample SC or extract can be negative relative to VC by random variation. If more negative than -10% RME2, anti-EA activity or toxicity would be suspected. Inclusion of an SC accounts for any extraneous residual EA that might exist in the media, extraction solution, or derived from the materials used for sample preparation. If the EA of the SC was greater than 15% RME2, then the entire experiment was rejected. The SDs for the extract or SC replicates for a given assay were typically so small that they fell within the space taken up by symbols used to plot the averages of %RME2 data for each concentration.

The greatest %RME2 response of 4–8 dilutions of a test chemical or extract run in triplicate was considered detectable if it produced an effect whose average %RME2 was greater than 15% RME2, i.e., more than three standard deviations (3SD, p <0.01, Student’s t test) above the mean SC response of a triplicate run for that particular assay*.* Such an “EA/no EA” categorical criterion/cut-off provides a rather conservative measure of detection for positive EA in an extract and has been previously used for detection of EA in plastics or other substances [16, 17, 22, 23, 27]. For known test chemicals assayed by the BG1Luc or MCF-7 assay (e.g. E2, TPP), the concentration that produced half-maximal stimulation by the test chemical (EC50, in M) was calculated from best sigmoidal fits to dose–response data using GraphPad Prism [16, 17, 23, 27].

Agonist stimulation of MCF-7 proliferation or BG1Luc Luciferase activity was confirmed to be estrogenic (versus non-specific) if the stimulation by a test chemical or extract was suppressed by co-incubation with the pure anti-estrogen ICI at 10^-7^ - 10^-8^ M [16, 17, 23, 24]. These *in vitro* “confirmation assays” rarely produce false positive responses [16, 17, 23, 27] and we saw no examples of an agonist response that was not suppressed by ICI in this study.

### Statistical comparisons

**Figure 2:**
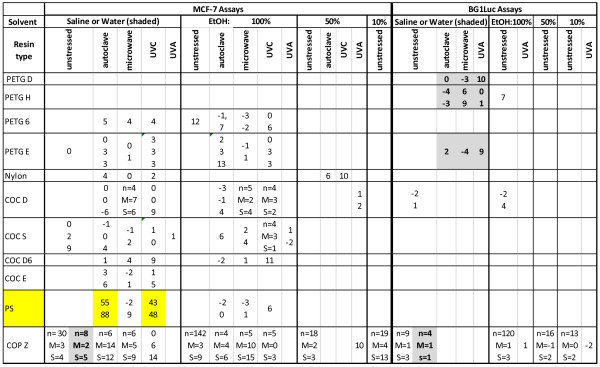
**EA (%RME2) of eleven BPA-replacement resins.** Each individual value is the maximum %RME2 that is the mean of 3–4 intra-assay data points for a single assay run on different wells of the same plate (see Methods). The mean (M) and SD (S) values of EA for a given type of assay in a single cell of the table is given when ≥4 independent assays were performed for that given assay condition, i.e., for the cell representing COC D, MCF-7 assay, saline extract, microwave stress. If no assays were run for the conditions associated with a cell in Figure 2, no EA values are given for that cell (i.e., the cell is blank). Many more assays were performed on COP Z resin cpompared to other resins because COP Z resin was used as a negative control in various assays for EA. When four our more individual assays were performed for the variables associated with a given cell, the number of independent assays (n), their mean (M) and their standard deviation (S) are indicated. Yellow highlighted cells, or combination of cells, indicate that at least 3 assays were consistently positive for EA, i.e., exhibited EA significantly greater (p <0.01, Student’s T-test, %RME2 ≥ 15) than VC and SC values (see Methods). A yellow-highlighted resin (e.g., PS) indicates a resin for which at least one cell or combination of cells exhibited EA. Grey-highlighted cells indicate assays using distilled water as an extract solution.

**Figure 3:**
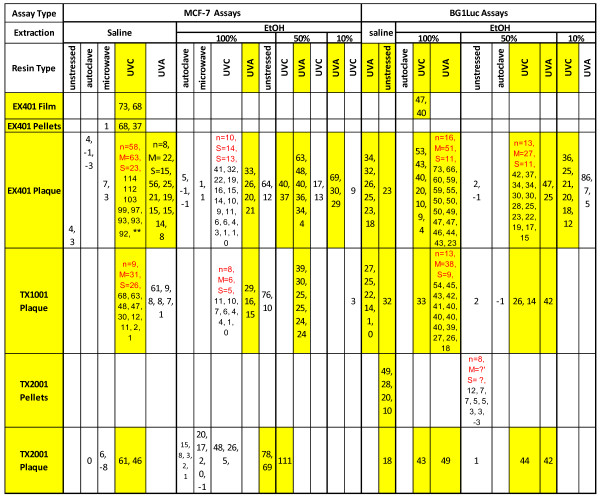
**EA (%RME2) of three Tritan™ BPA-replacement resins.** Each individual value is the maximum %RME2 that is the mean of 3–4 intra-assay data points for a single assay run on different wells of the same plate (see Methods). The mean (M) and SD (S) values of EA for a given type of assay in a single cell of the table is given when ≥8 independent assays were performed for that given assay condition, i.e., for the cell representing Tritan, MCF-7 assay, saline extract, UVA stress. If no assays were run for the conditions associated with a cell in Figure 2, no EA values are given for that cell, i.e., the cell is blank. Many more assays were performed on Tritan™ EX401 plaques, MCF-7 assay, saline extyract UVA stress compasred to other resins because Tritan™ EX401 resin was used as a positive control in various assays for EA. When eight our more individual assays were performed for the variables associated with a given cell, the number of independent assays (n), their mean (M) and their standard deviation (S) are indicated. Yellow highlighted cells, or combination of cells, indicate that at least 3 assays were consistently positive for EA (i.e., exhibited EA significantly greater (p <0.01, Student’s T-test, %RME2 ≥ 15) than VC and SC values (see Methods). A yellow-highlighted resin (e.g., all Tritans resins tested) indicates a resin for which at least one cell or combination of cells exhibited EA. **%RME2 values for 58 MCF-7 assays of Tritan™ EX401 stressed by UVC and extracted by saline: 146, 114, 112, 103, 99, 97, 93, 93, 92, 91, 91, 90, 90, 90, 88, 88, 88, 87, 86, 85, 85, 85, 80, 79, 72, 69, 69, 69, 69, 68, 67, 66, 66, 64, 64, 63, 58, 53, 53, 50, 49, 49, 47, 47, 45, 45, 45, 42, 41, 39. 39, 35, 16, 13, 9, 9, 8, 6.

Each assay using MCF-7 cell or BG1-Luc cells of a given test substance (e.g., TPP) or a given extract (e.g., 100% EtOH) of a given resin (e.g., Tritan EX401) exposed to a given stress (e.g., no stress, UVA, etc.) was run on a plate that had various dilutions of the test substance or extract in different wells and also had wells containing the VC or SC. Each test solution was repeated 3–4 times in different wells of the 96 well plate. The *intra-assay* variation and difference between the mean ± SD of the normalized %RME2 values of the extract (or the test substance) and the VC (or SC) were analyzed by Student’s T-Test with n equal to 3 or 4. In illustrations showing %RME2 values, a dotted line shows the 15%RME2 value that was always 3SD greater than the VC or SC values for each assay.When a given resin was extracted, stressed and run in the same manner on different days (e.g., a single cell in Figures 2 or 3), the mean ± SD of the %RME2 values of these “n” repeated assays of the same type were sometimes calculated. Figures 2 and 3 present the individual %RME2 values for such assays. When an assay of the same type was run on the same resin, but the stress condition was varied (e.g., microwave stress versus QUV stress in Figures 2 and 3), then the two means and SD’s of these two sets of repeated assays were sometimes compared using a two-tailed Student’s T-test.

When different assays were run on the same resin or class of closely related resins (e.g., three Tritan resins), the data were categorized as “yes” versus “no” for EA if the %RME2 for each individual assay was equal to, or greater than, 15%RME2 versus less than 15%RME2, respectively. Such “inter-assay” assessments of the probability of detecting significant amounts of EA were analyzed by Pearson’s Chi-Squared test with Yates correction for a 2×2 matrix (0 vs 14d) and by Pearson’s Chi-squared test for a 5×2 matrix (0, 1, 2, 7 and 14d). We performed all statistical analysis using GraphPad Prism5 (GraphPad Software, San Diego, CA) and/or R. Studio. All p-values were two-tailed.

The number and type of replications described above are indicated in each figure and table and/or its legend.

## Results

The relative estrogenic potency of chemical(s) extracted from thermoplastic resins was determined from concentration (dilution)-response analyses for BG1Luc and MCF-7 assays. These well-established bioassays measure the ability of chemicals to stimulate estrogen receptor (ER)-dependent induction of luciferase gene expression in BG1Luc cells [16] or proliferation of MCF-7 cells [1, 2, 16, 23, 28, 29]. All chemicals used to manufacture a plastic resin are almost never fully disclosed by the manufacturer who also typically does not know what additional chemicals might be generated by polymer synthesis at high temperatures (e.g., >230°C) and pressures. Both assays quantify the total EA of chemicals that leach from a plastic resin, but they do not identify the specific chemicals.

**Figure 4:**
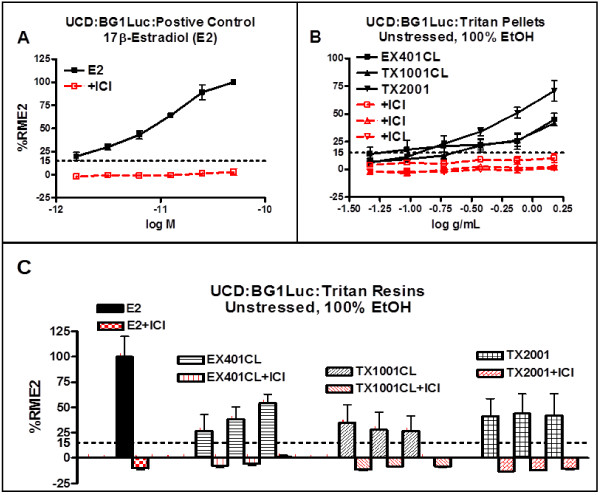
**Responses of BG1Luc cells at UCD to unstressed Tritan™ resin leachates.** Concentration-response curves for BG1Luc cells (UCD) incubated with the indicated concentration of **(A)** E2 (M) or **(B)** specific extract equivalents (g/ml) of three Tritan™ thermoplastic resins for 24 hours and luciferase activity determined. **(C)** The three Tritan™ thermoplastic resins were each extracted in intra-assay triplicate with 100% EtOH repeated on three separate days (i.e., three repeated assays of the same type on the same unstessed resin). The maximum Luciferase activity was determined for each of the three repeated assays and expressed as the %RME2 ± SD for each resin. Solid black lines and data points show agonist activity for all data points not associated with toxicity (see Methods); red lines and data points show results of coincubation of BG1Luc cells with E2 or the leachates and 10^-8^ M ICI. Horizontal dotted line shows the 15%RME2 value that is significantly (p <0.01, Student’s T-test) greater than the VC and SC (i.e. at least 3 SD greater). This is a very conservative criterion for declaring a positive agonist response.

**Figure 5:**
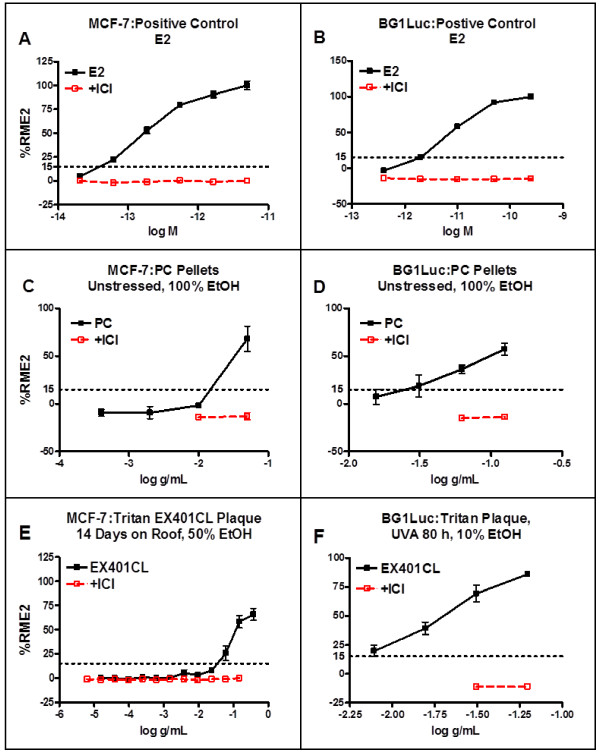
**Concentration-responses of BG1Luc and MCF7 cells at CCi to unstressed PC and stressed Tritan™ resin leachates.** BG1Luc and MCF7 cells at CCi were incubated with the indicated concentration of E2 **(A,B)**, unstressed PC pellet leachate **(C,D)** or stressed EX401 Tritan™ resin **(E, F)** in triplicate wells. Luciferase activity (BG1Luc cells) or proliferation (MCF7 cells) of intra-assay triplicates was determined as described under Materials and Methods and as described for BG1 cells in the legend for Figure 4. Concentration-response data are expressed as the mean ± SD of such triplicate analyses. Solid black lines and data points show agonist activity for all data points not associated with toxicity (see Methods); red lines and data points show results of coincubation of BG1Luc/MCF7 cells with E2 or extracts and 10^-8^ M ICI. Horizontal dotted line shows the 15%RME2 value that is significantly (p <0.01, Student’s T-test) greater than the VC and SC.

Figure 2 presents data obtained at CCi for the total EA (%RME2) of chemicals extracted from eleven BPA-free PC-replacement resins, stressed or unstressed, sorted first by type of assay, type of extract, and type of stress. Each %RME2 data point was calculated from concentration (dilution)-response curves for MCF-7 and BG1Luc assays, as previously reported (16,23,27) and as shown in Figures 4A,B and 5. The mean %RME2 and its standard deviation are given for any “cell” in Figure 2 for which at least four independent assays of the same type were run. Figure 3 presents data obtained at CCi for the total EA (%RME2) of chemicals extracted from the three unstressed and stressed Tritan™ resins, sorted first by type of assay, type of extract, and type of stress as described for Figure 2. The mean %RME2 and its standard deviation are given for all cells in which at least eight independent assays were run. Figure 3 is in a different format from Figure 3 because many more data were collected on Tritan resins than any other resin because of a previous report [30] that Tritan™ resins and products should not release chemicals having EA. A resin was considered “positive for EA” if it reliably exhibited EA at >15%RME2 for three assays in at least one assay condition or combination of assays of different extracts or stresses. Cells having at least three replication of the same assay type consistently showing EA, or combinations of three cells consistently showing EA, are highlighted in yellow in Figures 2 and 3. For example, MCF-7 assays of saline extracts of PS resins that were autoclaved (n = 2/2) or exposed to UVC (n = 2/2) consistently showed significant levels of EA (Figure 2), as did four BG1Luc assays of Tritan™ TX 2001 plaques extracted by EtOH and exposed to UVC or UVA (Figure 3).

We usually do not compare the relative level of EA measured as %RME2 released by different resins for comparative purposes unless the same assay and extract was used to assess that sample because: (1) The slopes of the extract dilution-response curve often cannot be confidently calculated (2) Extraction conditions were not all the same and different solvents may extract different amounts of different chemicals in the resin. (3) Chemicals having EA that are more volatile than the extraction solvent may be lost in vacuum extractions and EtOH is more volatile than water. (4) The sensitivity as defined by EC50 measures are not the same for the same chemical in different assays and the chemical(s) having EA in any extract are not known. (5) The total EA measured in any extract is a net activity of both the EA and anti-EA of all the chemicals present in the extract.

### Concentration-EA response analyses of thermoplastic resin extracts

Figure 4A,B shows concentration-response curves for the positive control (E2) and extracts of the three unstressed Tritan™ resins at UCD obtained using the UCD version of the BG1Luc assay. Each concentration of each extract or E2 is the intra-assay mean of triplicate wells (see Methods). Figure 4C plots these UCD data as the total maximum %RME2 value relative to the maximum value for E2 (%RME2) obtained for each concentration-response curve of each unstressed resin. Data points >15%RME2 were classified as EA positive. Extracts of all three unstressed Tritan™ resin plaques examined at UCD using the BG1Luc assay exhibited detectable release of chemicals having EA (Figure 4B,C).Similar dose–response data for E2, a PC extract and for extracts of stressed resins were obtained at CCi using the CCi version of the MCF-7 assay (Figure 5A,C,E) or the BG1Luc assay (Figure 5B,D,F). As described for BG1Luc assays at UCD, data points >15%RME2 were classified as EA positive for both MCF-7 and BG1-Luc assays at CCi. Figure 5 shows concentration-dependent increases in EA relative to SC at CCi for E2 and a PC extract in 100% EtOH using CCi’s MCF-7 (Figure 5A,C) or BG1Luc (Figure 5B,D) assay and two types of Tritan™ resin pellets stressed by natural sunlight (Figure 5E) for 14 days or by UVA for 80 hours (Figure 5F).

The ability of the pure ER antagonist ICI to inhibit the concentration-dependent induction of luciferase in BG1Luc assays and cell proliferation in MCF-7 assays produced by E2 and extracts of PC resins (Figures 4 and 5), and extracts of unstressed (Figure 4B,C) or stressed Tritan™ (Figure 5E,F) resins, demonstrated that these induction responses were all ER-dependent. In fact, we saw no example of an unsuppressed agonist response in this study, consistent with previous reports that very few agonist responses are not suppressed by ICI using either cell type [1, 2, 16, 17, 23, 26–28]. Data shown in Figure 5E also confirm that Tritan™ resins stressed by exposure to UV light (UVC, UVA or natural sunlight) released chemicals having EA.

### Survey of BPA-free thermoplastic resins for release of chemicals having EA

At CCi, we examined the ability of various extraction solvents (water, saline, and several concentrations of EtOH) to release chemicals with EA from fourteen different resins that were unstressed or stressed by autoclaving, microwaving, and/or UV irradiation. Extracts of unstressed COP Z, four COC, four PETG E, and one nylon resin had no detectable EA >15%RME2 or after any stress (Figure 2). In contrast, EA was detected with some, but not all, extracts of stressed PS (Figure 2) or three unstressed or stressed Tritan™ resins (Figure 3; Figure 5E,F).

Detection of significant levels of EA in extracts of all three *unstressed* Tritan™ resins in BG1Luc assays at UCD (Figure 4B,C), was particularly notable, especially given that Osimitz et al. [30] concluded that that (unstressed) Tritan™ resins would be expected to be EA-free, although these investigators never presented any actual results for Tritan™ resin extracts. For example, 3 unstressed pellets of each Tritan™ resin (EX401, TX1001, and TX2001) were extracted in triplicate with 100% EtOH on each of three separate days, and the EA of each of the nine extracts was determined in triplicate assays using the BG1Luc cells (a total of 27 separate assays). As shown in Figure 4C, extracts of all three unstressed Tritan™ resins consistently exhibited EA in every one of these assays, and the ability of ICI to block the induction response indicated the role of the ER in the positive EA response. Thus, our data from the BG1Luc assay at UCD clearly demonstrated that unstressed Tritan™ EX401, TX1001 and TX2001 resins contain extractable chemicals with detectable levels of EA. BG1Luc assays of Tritan™ resins extracted by saline at CCi also consistently (7/8) exhibited detectable levels of EA (Figure 3) but not by 50% EtOH (0/12). This difference could be an inherent difference between the ability of the two solvents to extract chemicals with EA leaching from Tritan™. Similarly, the relatively few MCF-7 assays of unstressed Tritan™ at CCi using saline (0/2) did not detect significant release of EA, but 4/6 assays using 50% EtOH did detect significant levels of EA.

As stated above, if EA was detected three times in any solvent extract (or combination of extracts) from any resin in either assay, that resin was classified as leaching chemicals having EA and is highlighted in yellow in Figures 2 and 3. Although the analyses of the various extracts using the BG1Luc and MCF-7 assays produced slightly different %RME2 values (Figures 2 and 3), the overall results of each assay led to the same conclusion that a given resin did or did not release chemicals having detectable EA. For example PETG, nylon, COC and COP Z resins did not release chemicals having significantly detectable levels of EA under any conditions tested. PS resins released chemicals having detectable levels of EA when extracted with saline. Detection of significant (>15%RME2) levels of EA in some, but not all extracts, of different resins of the same type (e.g., different Tritan™ resins in Figure 3) was not surprising, and could result from different additives and impurities (catalyst residues, thermal degradation products, etc.) in the different resins and/or differences in their processing [16, 17].

Although EA was detected in all solvent extracts of three stressed Tritan™ resins using both assays (Figure 5; Figure 3), Tritan™ resins stressed with two forms of UV radiation (UVC, UVA) rather consistently released detectable levels of EA (>15%RME2), as indicated by the pattern of yellow highlighted cells in Figure 3, especially when compared to resins stressed by autoclaving or microwaving. For example, for EX401 resins, 0/3 MCF-7 assays in saline extracts showed significant EA when stressed with autoclaving, but 53/58 when stressed with UVC and 6/8 when stressed with UVA. Similarly, when this resin was extracted with 100% EtOH, chemicals having significant levels of EA were consistently released when this resin was stressed with UVA (4/4) in MCF-7 assays, but not when autoclaved (0/3). The levels of EA released were also higher in MCF7 assays when this resin was UV stressed compared to autoclaving. For example, the mean ± SD %RME2 of Tritan™ resin EX401 extracted with saline and stressed with UVC (n = 58, 63 ± 23%) was significantly (p = 0.0017, Student’s T-test, two tailed) higher versus stress by autoclaving (n = 3, 0 ± 3%). When saline extracts of EX401 were stressed with UVA, the EA level (n = 8, 22 ± 15%RME2) was also significantly (p = 0.032, Student’s T-test) higher compared to stress by autoclaving. When exposed to UVA and extracted with 100% EtOH, the level of EA (25 ± 6%RME2) was also significantly higher (p = 0.0016, Student’s T-test) than when autoclaved (1 ± 3.5%RME2). These data also showed that stressing *per se* (i.e., autoclaving or microwaving) did not necessarily cause release of chemicals having significant EA from Tritan™ resins.

### UV radiation penetrates thermoplastic resins

**Figure 6:**
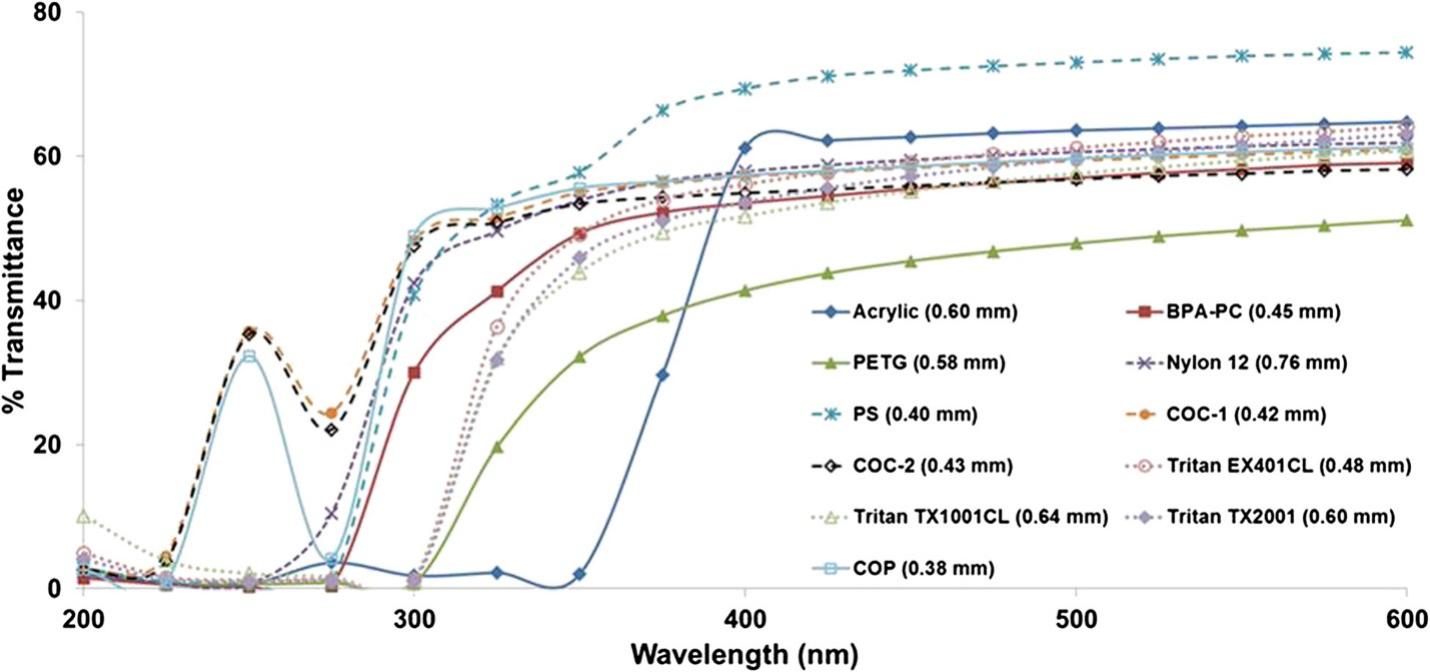
**UV/Visible spectra of PC-replacement thermoplastic resins.** UV/Visible spectra of various PC-replacement thermoplastic resins of stated thickness (mm) showing percent light transmittance at different wavelengths (nm). EA of thermoplastic resins can result from exposure to UV light or inclusion of an estrogenic chemical in their manufacture.

**Figure 7:**
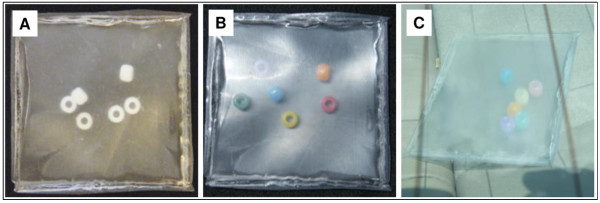
**UV-detecting beads change color when sunlight passes through Tritan™ TX2001 plaques.** Demonstration that sunlight can pass through a sealed pouch formed from Tritan™ TX2001 to cause the UV-detecting beads to change color. **A**. Unexposed beads in Tritan pouch. **B**. Beads in Tritan pouch exposed to natural sunlight. **C**. Beads in Tritan pouch exposed to natural sunlight through a window. A color change in the beads shows that UV radiation has been captured by a bead. The change to one color versus another color has no significance.

The results described above revealed that exposure to UV radiation can increase the EA of extracts from three Tritan™ thermoplastic resins assayed in this study. UV light in the presence of oxygen can degrade thermoplastic polymers [31] and thus could contribute to EA release. However, we often extract from the outer and inner surfaces of plaques. UV radiation might affect only the outer surface [31] of a plastic resin and UV wavelengths might not penetrate these thermoplastic resins to any significant degree.To examine whether UV light might increase the leaching of chemicals having EA from the inner surface of a resin, we first determined the UV/Visible spectra of compression-molded plaques of similar thickness made from three Tritan™ or eight other thermoplastic resins (Figure 6). These results demonstrated that UV can completely penetrate thermoplastic resin plaques. The ability of UV light to penetrate these thermoplastic resins was visually confirmed in experiments in which UV-detecting beads that change from white to a color when exposed to UV radiation in the range of 300–360 nm were inserted into a sealed pouch made from Tritan™ TX2001 plaques (see Methods). The beads in the pouch were white prior to UV exposure (Figure 7A). The beads changed to a color when exposed to sunlight on a roof or even through the rear window of a car (Figure 7B,C). These results demonstrated that UV radiation could degrade PC-replacement thermoplastics on both the outer and inner surfaces of a plaque or product made from such materials, thereby potentially producing and/or releasing chemicals having EA into solutions contained within a product.

### Natural sunlight can increase release of chemicals having EA

**Figure 8:**
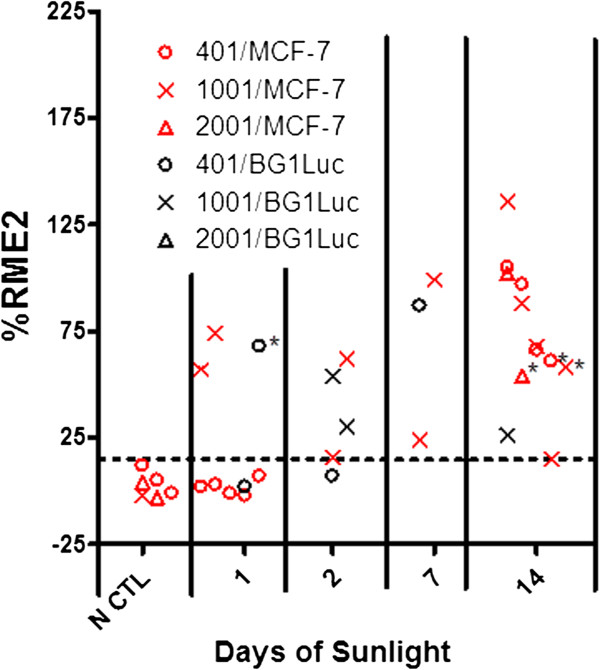
**Exposure to natural sunlight increases probability of release of chemicals having EA from Tritan™ resins.** EA of ethanol extracts of Tritan™ resin plaques that were not exposed (N CTL) or exposed to sunlight for up to 14 days. Values for EA were determined using the MCF7 assay. The dotted line in the panel equals 15%RME2, a value that is significantly (p <0.01, Students T-test) greater than the vehicle control for each assay (i.e., intra-assay triplicate values). For inter-assay comparisons using Chi-Squared analyses (See Methods), plaques having EA equal to or greater than 15%RME2 were defined as exhibiting significant levels of EA; plaques having EA less than 15%RME2 were defined as not exhibiting significant lecvels of EA. That is, the EA of these plaques was treated as a yes/no categorization. Most resins were extracted using 50% EtOH as the solvent; four resins (indicated by a black asterisk) were extracted using 100% EtOH as a solvent.

To examine the effect of natural sunlight exposure on release of chemicals with EA from a thermoplastic resin, we determined the effect of sunlight on compression-molded Tritan™ plaques that had shown significant release of EA when exposed to artificially-generated UVA or UVC (Figures 2 and 3 and Figure 5), as described above. The resin plaques were exposed to natural sunlight by placing the plaques between a quartz glass plate and aluminum foil-lined ceramic tile. Quartz glass was used because it allowed more UV radiation to pass completely through (http://www.rayotek.com/technical_info_glass_sapphire.htm) compared to soda lime window glass. To control for heat versus sunlight effects, Tritan™ plaques were fully wrapped in aluminum foil to avoid exposure to sunlight and were kept on the roof for up to 14 days (negative controls). Other Tritan™ plaques were exposed to natural sunlight for 1,2, 7 or 14 days. All resins were extracted by 50% EtOH or 100% EtOH (asterisks in Figure 8) and tested at CCi for EA by the MCF-7 assay (red symbols in Figure 8) or by the BG1Luc assay (black symbols in Figure 8).As illustrated in Figure 8 considering all Tritan™ resins as a family of similar resins, the observed proportion of obtaining EA positive samples from any Tritan™ resin increased from 0/6 control resins not exposed to sunlight (0 days in Figure 8) to 12/12 resins exposed to sunlight for 14 days. This increased probability of obtaining positive EA samples was significantly higher (p = 0.0015, Pearson’s two-tailed Chi Squared test with Yates correction) for the 12 Tritan™ plaques exposed to sunlight for 14 days compared to the 6 negative controls. Increases in days of exposure to sunlight from 0,1, 2, 7 to 14 days significantly (p <0.001, Pearson’s Chi-Squared test for a 5×2 matrix) increased the probability that a Tritan™ resin would be classified as positive for EA release. These agonist responses for EA with exposure to sunlight were suppressed by ICI, as shown in Figure 5E.

### TPP, an additive for some resins, has EA

**Figure 9:**
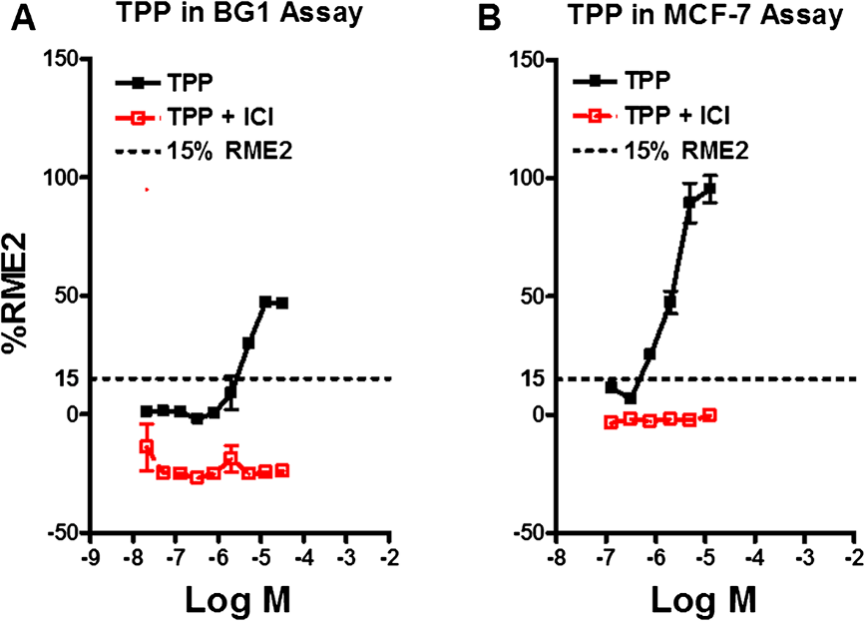
**TPP exhibits EA in a concentration–dependent manner.** BG1Luc **(A)** or MCF-7 **(B)** cells were incubated with increasing concentrations of TPP and EA at CCi and EA determined as described under Material and Methods. Values are the mean ± SD of three wells (intra-assay “triplicates”) containing the same solution and are expressed as a percent of the maximal activity produced by E2 (%RME2). Solid black lines and data points show agonist activity for all data points not associated with toxicity (see Methods); red lines and data points show results of co-incubation of BG1Luc/MCF7 cells with TPP and 10^-8^ M ICI. Horizontal dotted line shows the 15%RME2 value that is significantly (p <0.01, Student’s T-test) greater than the VC and SC.

The results presented above indicated that three unstressed and stressed Tritan™ resins released chemicals with EA. Although the chemical(s) responsible for the EA remain to be determined, Tritan™ resins are known to be manufactured using the additive TPP [32] and a previous study has reported that TPP has EA [33]. To determine whether TPP could contribute to the EA detectable in extracts of Tritan™ resin in our assays, concentration-EA response studies for TPP were carried out using BG1Luc and MCF-7 assays at CCi. Figure 9 clearly shows that TPP exhibits significant EA in both BG1Luc and MCF-7 assays (p <0.01, Student’s T-test), with an EC_50_ of 4.7×10^-6^ and 2.2 × 10^-6^ M, respectively, and these positive responses were inhibited by ICI. Although TPP has EA in our assays and as previously reported [17], its relative contribution to the overall EA of extracts of Tritan™ resins remains to be confirmed.

## Discussion

### Release of chemicals having EA from BPAreplacement thermoplastic resins

While the release of chemicals from plastic resins or products has been well studied by a number of laboratories [20, 21], the leaching of chemicals with EA from many different unstressed or stressed plastic products has only recently been examined [16, 17, 34, 35]. BPA is by far the best studied of the estrogenic chemicals released from PC plastics (for reviews see 4,10,11,36]. The identification of BPA in plastics coupled with its EA and potential to produce adverse health effects, particularly in developing embryos and newborns, led to a public outcry and subsequent elimination of BPA from many plastic products and a ban of BPA in plastic products for babies by the US FDA and the European Union [13]. However, in addition to BPA, other chemicals with EA are utilized in the preparation of plastic resins [4, 8, 9, 16, 17].

In the first extensive study of BPA-free plastics that existed on the market from 2005–2009 using only the MCF-7 assay, Yang et al. [16] reported that chemicals exhibiting EA were released from 0%-100% (depending on which extraction solvent was used). That number increased to 50-100% (average of 72% overall) if one or more solvents were used for each sample of 455 unstressed plastic products made from 6 identified BPA-free resins. [Only one resin, PS, was a PC-replacement resin]. The number of unstressed plastic products releasing chemicals having EA increased to 92% if two extraction solvents (saline or EtOH were used). Only a few products were examined after receiving any stress (see Figure five of ref [16]). More recently, others have reported release of chemicals having EA in unstressed PET plastics [36] or that BPS and a few other chemicals used to make some BPA-replacement plastics exhibit EA [14, 15, 36]. Most recently, we [17] examined 50 BPA-free PC-replacement plastic products using two assays (MCF-7 and BG1Luc), various extraction solvents and stress conditions. Most products made from acrylic, polyethersulfone (PES), PS and Tritan™ resins exhibited EA, but most products made from COC, COP or PETG resins did not release any chemicals having significant (detectable) levels of EA [17]. Exposure to UVA and UVC significantly increased the probability of release of chemicals with EA from these BPA-free replacement products (Figures two and three of [17]). However, none of these previous studies identified the possible origin for the release of chemicals having EA at the most basic level, i.e., the BPA-replacement resin itself or chemicals added to the BPA-free resin during the processes used to manufacture the finished product (Figure 1).

Accordingly for the data reported herein, using two mammalian cell-based assays and several extraction procedures, we investigated whether some unstressed and variously-stressed BPA-free, PC-replacement resins released chemicals having EA. Data from more than 600 assays showed that some (4/14) PC-replacement thermoplastic resins, including a PS resin and all three Tritan™ resins tested, leached chemicals having EA, irrespective of whether these resins were unstressed or stressed. Agonist responses obtained using BG1Luc or MCF-7 assays were always inhibited by ICI, confirming that agonist responses were due to ER activation.

Equally importantly, our current data also demonstrated that some (10/14) unstressed and stressed thermoplastic resins (four COC, one COP, one nylon, and four PETG) did not release chemicals having significant (≥15%RME2) levels of EA (Figure 2), indicating that it is possible to produce EA-free thermoplastic resins for commercial use. The resin data in this paper are consistent with data in [17] showing that products that did not release chemicals having detectable EA could be made from COC, COP, and PETG resins [17], presumeably because chemicals having EA were not added or produced during the manufacturing processes. We noted no consistent cost differential between PC-replacement resins having or lacking EA assayed in this paper, or for PC-replacement products made from such resins analyzed in our previous paper [17].

We emphasize that the results for the EA-free replacement resins reported herein do not imply that products made from such resins will *always* be EA-free, but rather they *can* be EA-free if the apprropriate additives and manufacturing processes are used. Furthermore, all resins of a given type (e.g., COC) are *not* sythetized using the same chemicals or processing protocols (See Figure 1) and therefore are *not* uniformly EA- free. For example, we also tested a COC resin (COC “F”) used to make films that are not re-usable and hence the resin was not listed in Figure 2. However, this COC F resin was not EA-free when extracted five times (n = 5) with saline or EtOH and microwaved (%RME2 16 ± 25 and 32% ±42, respectively) or exposed to UVC (%RME2 32 ± 40 and 32 ± 43, respectively). [The data included some %RME2 values at ~15% and some much higher, thereby giving a mean >15% and a high SD. In each case 5/5 or 4/5 %RME2 values were greater than 15%.] This COC F resin (n =5 samples) did not release chemicals having EA when autoclaved and similarly extracted (%RME2 of 7 ± 8 and 4 ± 9, respectively).

Although there are no standards yet proposed, much less adopted, to assess the EA in plastic resins or products, our data in this paper and in our previous papers [16, 17] clearly show that such regulations should include more than one extraction solvent to better detect hydrophilic versus hydrophobic chemicals in leachates. Furthermore, results from the current paper and from [17] show that various stresses that simulate some aspects of common use of plastics must also be assessed before reporting that a resin or product is EA-free. If only a single protocol were required to conclude whether a resin (or product) is EA-free, then that requirement would most-certainly lead to many false negative conclusions. For example, if the COC F resin described above were only tested with autoclave stress, it might well be declared to be EA-free, as might some PS or Tritan™ resins if assayed only after autoclaving or microwaving and extracted with only one solvent (see Figures 2 and 3).

### UV radiation can increase the probability of leaching of chemicals having EA

Exposure to UV radiation (UVC, UVA, and natural sunlight) increased the probability that chemicals having detectable levels of EA leached from Tritan™ resins compared to various combinations of unstressed, autoclave and/or microwave stresses. When it occurs, this increase in activity could result from UV-dependent formation of a chemical(s) with EA and/or enhanced release of a chemical(s) with EA. Although some UV wavelengths that travel through these resins are absorbed, some clearly penetrate to the inner surface of the resin (Figures 6, 7 and 8), Such UV radiation can produce chain scission and other chemical reactions in the presence of oxygen [37] that can increase the release of various substances from the resin, some of which could have EA. These data are consistent with previous reports [38] that exposure of Tritan™ to UV radiation causes degradation of the resin as evidenced by color changes, increased brittleness, and other physical changes and that UV radiation increases the probability that significant levels of EA can be released from products made from Tritan™ [16, 17].

Exposure to UV radiation is part of the aging process for many resins used to make reusable BPA-free plastic products. Our data showed that all forms of UV radiation tested (UVA, UVC and natural sunlight) typically increased the probability of detecting and/or the level of EA release. However, aging or stress per se did not always increase release of chemicals having EA from PC-replacement resins. For example, we often (but not always) found less release of chemicals for some resins having EA after autoclaving.

### TPP used in polymerization processes has EA

TPP exhibited detectable EA in both MCF-7 and BG1Luc assays (Figure 9), consistent with a report [33] showing that TPP can activate human ERα- and ERβ-dependent reporter gene expression. Since TPP can also antagonize the androgen receptor [33], TPP is both estrogenic and antiandrogenic. The estimated daily intake of TPP in food after contact with Tritan™ copolymer, which uses TPP in the polymerization process, is 1.5 × 10^-6^ mg/kg/day [32]. Furthermore, some of the decomposition products of TPP are phenols [39] and many phenolic compounds have EA [1–3, 16, 23, 27]. While some of the EA detected in Tritan™ extracts could arise from TPP and/or its breakdown products, this remains to be confirmed by instrumental analyses of the extracts.

### Assessments of EA from other studies of plastic resins

Our data demonstrating that unstressed Tritan™ leaches chemicals with EA are consistent with the more-limited set of previous data showing that four water bottles and a baby bottle made from Tritan™ resins release chemicals having EA, especially when stressed with UVC radiation (identified as PETG resins in Figure five of [16]). Our data are also consistent with data reported in a recent study [17] that products made from Titan™ resins almost always (23/25 products) released chemicals having EA, as did all (9/9) products made from PS resins exposed to UV. The two exceptions were two types of green bottles made from Tritan™ for which the particular green colorant(s) used during manufacture blocked the penetration of UV radiation [17]. In contrast, many products made from COC (2/2), COP (1/1), and PETG (2/3) resins did not release chemicals having significantly-detectable levels of EA (Figure two in [17]). That is, if a resin releases chemicals having EA, then the product will almost-certainly release chemicals exhibiting EA.

Our data demonstrating that unstressed Tritan™ leaches chemicals with EA are *not* consistent with a conclusion reported in a recent study by Osimitz et al. [30] that Titan™ resins should not release chemicals having EA because the three monomers (dimethyl-terephthalate (DMT), 1,4-cyclohexanedimethanol (CHDM), and 2,2,4,4-tetramethyl-1,3-cyclobutanediol (TMCD)) used in various ratios by Eastman Chemical to manufacture various Tritan™ resins purportedly had no detectable EA. In this Eastman-funded study, a combination of *in silico*, *in vitro* and *in vivo* methods were reportedly used to examine the estrogenic and androgenic activity of DMT, CHDM and TMCD. Although the lack of positive EA results reported for the three monomers in their yeast cell ER-transactivation bioassay [30] were interpreted to mean that these monomers have no EA, yeast bioassays have low sensitivity, a high rate of false negative results, and often do not respond appropriately to some ER ligands/antagonists [40]. Accordingly, while positive EA results from yeast assays are generally acceptable, negative EA results do not provide meaningful evidence that a test substance lacks EA [1, 2, 40]. Additionally, while Osimitz et al. [30] reported that the three Tritan™ monomers were inactive in ER-transactivation assays in recombinant human T47D cells, they only reported ER-transactivation results for CHDM, and these results actually revealed a CHDM concentration-dependent enhancement of E2-dependent luciferase gene expression; the effect of CHDM alone on ER-dependent gene expression was not shown. Furthermore, chemicals other than the three tested monomers are used in the manufacture of Tritan™ resins [18, 19, 32], and at least one of them (TPP) has EA ([33]; Figure 9).

### No *in vivo*assay data is available for resin EA

The results presented in this study and other described above examine the EA of extracts of plastic products utilizing a variety of *in vitro* cell-based assays. There are no studies of which we are aware examining the EA of extracts of any BPA-free PC-replacement resin (or product) using an *in vivo* assay (i.e. measurement of the ability of an extract to stimulate uterine growth or ER-dependent gene expression). Osimitz et al. [30] reported that a very dilute mixture of the three monomers that comprise part of the chemical composition of Tritan™ showed no EA in an uterotrophic assay and concluded that the manufactured resin should therefore leach no chemicals having EA. There were major problems with the experimental design of this in vivo study, including: (**1)** They evaluated the EA of the mixture of the three monomers at dosages below their no observable effect level (NOEL) instead of their maximum tolerated dose as recommended by OECD [41] and is standard practice in the field [42]. Using their protocol, E2 would be expected to have no EA. (**2**) They utilized an insensitive strain of rat for these studies [10], and (**3**) They tested only three ingredients of Tritan™ resins instead of all of the 8–10 chemicals that go into the preparation of the resins. **(4)** They never tested extracts of the resin which could release additional chemicals that could form during the high pressures and temperatures experienced polymer synthesis. No effort to identify such chemicals has been reported. Therefore, the negative *in vivo* test results obtained by Osimitz and coworkers [30] using only a mixture of three pure monomers provide little meaningful information as to whether extracts of unstressed or stressed resins would release chemicals having EA [42].

## Conclusions

In this survey of PC-replacement resins, we recognize that we quantify the maximum effects of total EA (%RME2) in extracts relative to the maximum effect of E2 using two sensitive assays, at least six extraction protocols, and at least six stress protocols. We define a resin as releasing chemicals having EA if an EA value >15%RME2 is observed in at least one assay condition. In fact for the resins reported in this paper, resins labeled as EA positive had at least three independent assays greater than 15% RME2 for a given assay condition and others at least three consistently-positive in at least three similar assay conditions (yellow-highlighted cells in Figures 2 and 3). Therefore, the current paper is an *in vitro* study that reports the existence of a possible hazard for consumption of chemicals with EA leaching from plastic products made from PS and Tritan™ resins that leach chemicals having detectable levels of EA.

This study does not assess the risk that such consumption might have for human health. In fact, we believe that this risk cannot be adequately assessed at this time because neither we nor any other scientists or entity to our knowledge have identified and characterized the EA and anti-EA of all chemicals and their metabolites in these extracts, including those formed during different stresses. How much of the total EA that leach from plastics made from these resins or other sources is consumed and absorbed by human subjects is another unknown as are short-term and long-term effects at different life stages (fetus, infant juvenile, adult, etc.). These are areas that need in depth analyses and evaluation by scientists or entities that do not have a financial or ideological stake in a particular set of results.

Nevertheless, the results of our potential-hazard study are important because other studies have reported that chemicals with EA in mammals can produce various adverse health effects such as early menarche, reduced sperm counts and other altered functions of reproductive organs, obesity, and increased rates of some cancers. Some of these effects occur at very low doses in fetal, infant, and juveniles, but are often only detected in the adult stage [4, 8, 11]. These animal studies are relevant to humans [12], as dramatically and unfortunately demonstrated by the adverse health effects on the offspring of mothers given diethylstilbestrol, a chemical exhibiting high EA [3, 4, 8, 10, 11, 36].

In summary, our MCF-7 and BG1Luc assays demonstrate that extracts of four unstressed and/or stressed BPA-free thermoplastic resins, one PS and three Tritan™ resins, release chemicals that can activate ER-dependent cell signaling. These data and our conclusion on Tritan™ resins reported herein, combined with data for those products assessed in [17], are in stark contrast to those of Osimitz et al. [30], whose data are not relevant to assess the EA in extracts of Tritan™ resin. Considering all the available data, we conclude that these four BPA-free thermoplastic resins are not EA-free. This conclusion is especially important because our data on products made from other BPA-free, BPA-replacement resins assayed in a related survey study [17], show that it is possible to synthesize thermoplastic resins in commercial quantities that are usable to manufacture hard and clear products that could be EA-free, assuming that chemicals added, used, or created in the manufacturing process are also EA-free [16, 17]. Given that plastic products have advantages (weight, cost, impact strength, energy footprint, etc.) in various combinations compared to other materials such as steel or glass, our data suggest that these advantages of plastics can be maintained while avoiding potential adverse health effects of release of chemicals having EA into foodstuffs or the environment.

## Abbreviations

%RME2: Percent maximum response relative to the maximum response of 17β-estradiol (E2)
AU: Autoclave
BPA: Bisphenol A
BPS: Bisphenol S
CHDM: 1,4-cyclohexanedimethanol
CCi: CertiChem, Inc.
COC: Cyclic olefin copolymer
COP: Cyclic olefin polymer
DMSO: Dimethylsulfoxide
DMT: Dimethyl-terephthalate
E2: 17β-estradiol
EA: Estrogenic activity
EA-free: No detectable estrogenic activity
EC50: Half-maximal response
EFM: EA-free medium
ER: Estrogen receptor
EtOH: Ethanol
ICCVAM: Interagency Coordinating Committee on the Validation of Alternative Methods
ICI: ICI 182,780
MI: Microwave radiation
NICEATM: National Toxicology Program’s Interagency Center for the Evaluation of Alternative Toxicological Methods
NIEHS: National Institute of Environmental Health Sciences
NIH: National Institutes of Health
NSF: National Science Foundation
OECD: Organization for Economic Cooperation and Development
PC: Polycarbonate resin or product synthesized using BPA as a monomer
PE: Polyethylene
PETG: Glycol-modified polyethylene terephthalate
PES: Polyethersulfone
PPi: PlastiPure, Inc
PS: Polystyrene
RMPI: Roswell Park Memorial Institute
SC: Sham control aka method blank
TMCD: 2,2,4,4-tetramethyl-1,3-cyclobutanediol
TPP: Triphenyl phosphate
UCD: University of California, Davis
UV: Ultraviolet
UVA: 315–400 nm long wavelength UV
UVB: 280–315 nm medium wavelength UV
UVC: 180-280 nm short wavelength UV
VC: Vehicle control.
